# Serotonin Transporter Gene (*SLC6A4*) Variations Are Associated with Poor Survival in Colorectal Cancer Patients

**DOI:** 10.1371/journal.pone.0038953

**Published:** 2012-07-24

**Authors:** Sevtap Savas, Angela Hyde, Susan N. Stuckless, Patrick Parfrey, H. Banfield Younghusband, Roger Green

**Affiliations:** 1 Discipline of Genetics, Faculty of Medicine, Memorial University of Newfoundland, St. John’s, Newfoundland and Labrador, Canada; 2 Discipline of Oncology, Faculty of Medicine, Memorial University of Newfoundland, St. John’s, Newfoundland and Labrador, Canada; 3 Clinical Epidemiology Unit, Faculty of Medicine, Memorial University of Newfoundland, St. John’s, Newfoundland and Labrador, Canada; Klinikum rechts der Isar der TU München, Germany

## Abstract

Prognosis in colorectal cancer patients is quite variable, even after adjustment for clinical parameters such as disease stage and microsatellite instability status. It is possible that the psychological distress experienced by patients, including anxiety and depression, may be correlated with poor prognosis. In the present study, we hypothesize that genetic variations within three genes biologically linked to the stress response, namely serotonin transporter (*SLC6A4*), brain-derived neurotrophic factor (*BDNF*), and arginine vasopressin receptor (*AVPR1B*) genes are associated with prognosis in colorectal cancer patients. We used a population-based cohort of 280 patients who were followed for up to 12.5 years after diagnosis. Our multivariate analysis showed that a tagSNP in the *SLC6A4* gene (rs12150214) was a predictor of shorter overall survival (HR: 1.572, 95%CI: 1.142–2.164, p = 0.005) independent of stage, age, grade and MSI status. Additionally, a multivariate analysis using the combined genotypes of three polymorphisms in this gene demonstrated that the presence of any of the minor alleles at these polymorphic loci was an independent predictor of both shorter overall survival (HR: 1.631, 95%CI: 1.190–2.236, p = 0.002) and shorter disease specific survival (HR: 1.691, 95%CI: 1.138–2.512, p = 0.009). The 5-HTT protein coded by the *SLC6A4* gene has also been implicated in inflammation. While our results remain to be replicated in other patient cohorts, we suggest that the genetic variations in the *SLC6A4* gene contribute to poor survival in colorectal cancer patients.

## Introduction

Colorectal cancer is a common disease with millions of new cases worldwide each year. Although outcomes in patients have improved in the recent decades, because of earlier detection, improvement in diagnostic procedures, and development of better treatment options, current 5-years survival rates for the patients are quite disappointing: ∼60–65% in North America [1] and much lower in developing countries [2]. Therefore, similar to other cancers, there is a need to develop better strategies to clinically manage this disease.

Clinical outcomes are associated with disease stage, age, and co-morbidities [3]. But, patient outcome may be modified by other factors, such as emotional health and quality of life. An important determinant of the emotional health and quality of life in cancer patients is the patients’ effectiveness of coping with the psychological distress caused by the cancer diagnosis and the undesirable consequences of treatment. In patients with advanced stage, especially in palliative care, coping with the prospect of death is also a serious emotional burden. These challenges often surface as depression or anxiety in cancer patients, which are observed in up to 50% of cancer patients [4]. Some studies have suggested an association between distress coping effectiveness and survival in cancer patients [5], [6], although conflicting results have also been reported [7].

**Table 1:** Genes and SNPs investigated in this study.

Gene	SNP ID	Polymorphism	MAF	Patients with available genotypes
*SLC6A4*	rs4251417	G>A (non-coding)	8%	272 (97.0%)
*SLC6A4*	rs12150214	G>C (non-coding)	39.30%	271 (96.8%)
*SLC6A4*	rs140700	G>A (non-coding)	9.30%	268 (95.7%)
*BDNF*	rs6265	G>A (Val66Met)	19.40%	271 (96.8%)
*AVPR1B*	rs35369693	G>C (Lys65Asn)	6.80%	264 (94.3%)

Summary of the genetic variations included in this study. **MAF:** minor allele frequency, **SNP ID:** dbSNP database [41] SNP identifiers.

Susceptibility to depression or anxiety has been linked to several genes, including, *SLC6A4*, which codes for the serotonin transporter protein (also known as 5-HTT and SERT). Serotonin is a neurotransmitter, deficiency of which leads to clinical depression in susceptible individuals [8]. By regulating the serotonin uptake and its synaptic availability, 5-HTT has an important role in serotonin metabolism. Various observational and experimental studies have shown that *SLC6A4* and its variations are involved in susceptibility to several psychiatric conditions in humans and mice, including depression and anxiety [8]–[10]. The *BDNF* gene codes for a protein (brain-derived neurotrophic factor) involved in survival, growth, and differentiation of neurons [11], and, like *SLC6A4*, is involved in the response to stress. Several studies, including those in *BDNF*-mutant mice, have suggested that alterations in expression or function of this gene are linked to anxiety, depression and post-traumatic disorder [12], [13]. Another gene involved in stress response is the *AVPR1B* gene coding for the arginine vasopressin receptor [14]. Functional and epidemiological studies have shown that this gene and its ligand vasopressin are also linked to anxiety and depression in humans and in model species [15], [16].

In this study, we hypothesized that genetic variants located within the *SLC6A4*, *BDNF*, and *AVPR1B* genes are associated with prognosis in colorectal cancer patients. Therefore, we tested the association of five such variations from these genes with survival in a colorectal cancer cohort from Newfoundland, Canada.

## Materials and Methods

### Ethics Statement

This study was approved by the Human Investigation Committee of the Memorial University of Newfoundland as well as the Regional Health Boards. Informed consent was not required by the local ethics board as the study was considered an anonymous chart review. Patient data was analyzed anonymously.

**Table 2:** Baseline characteristics of the patient cohort.

Variables	n	%
**Sex**		
male	150	53.57
female	130	46.43
**Median age at diagnosis**	68.4 years,range (25.3–91.6)	
**Grade**		
poorly differentiated/undifferentiated	42	15.0
well/moderately differentiated	234	83.6
unknown	4	1.4
**Histology**		
mucinous	43	15.4
non-mucinous	237	84.6
**Location**		
rectum	57	20.4
colon	223	79.6
**Stage**		
I	54	19.3
II	94	33.6
III	76	27.1
IV	47	16.8
unknown	9	3.2
**MSI status**		
MSI-H	34	12.1
MSI-L/MSS	246	87.9
**OS status**		
dead	172	61.4
alive	108	38.6
**Median OS and DSS** **(follow up) time**	5.30 years,range (0–12.5 years)	
**DFS status**		
recurrence/metastasis/death (+)	184	65.7
recurrence/metastasis/death (−)	96	34.3
**DFS (follow up) time**	3.37 years,range (0–12.5 years)	
**DSS status**		
death from colorectal cancer	113	40.4
death from other causes/alive	167	59.6

Summary of the baseline characteristics of the study cohort. **(+):** present, **(−):** absent, **DFS:** disease-free survival, **DSS:** disease-specific survival, **MSI-H:** microsatellite instability-high, **MSI-L:** microsatellite instability-low, **MSS:** microsatellite stable, **n:** number of samples included into the analysis, **OS:** overall survival.

### Patient Cohort

This is a population based and retrospective study. From the Avalon Peninsula of Newfoundland, new cases (diagnosed at the Health Sciences Centre, Grace General Hospital, St. Clare’s Mercy Hospital (all in St. John’s), and Carbonear General Hospital) were ascertained over a two-year period between January 1, 1997 and December 31, 1998. Surgical specimens were available for 280 of the 292 patients identified. DNA was extracted from formalin-fixed paraffin-embedded non-tumor colon and rectum tissues. Outcomes were ascertained using medical records until July 2009.

### Selection and Genotyping of Polymorphisms

The three genes (*SLC6A4*, *BDNF* and *AVPR1B)* were prioritized and investigated in this study because of their biological roles in depression or anxiety. We identified tagSNPs with a minor allele frequencies >10% in these genes using the genotype information for Caucasians posted in the HapMap database, phase I & II (http://hapmap.ncbi.nlm.nih.gov/) and using the pair-wise tagger function implemented in the Haploview program [17]. Following this approach, we initially selected five tagSNPs in *SLC6A4* (rs11080121, rs12150214, rs2066713, rs4251417, and rs140700) and two tagSNPs in *BDNF* (rs7124442 and rs6265). For *AVPR1B* we analyzed a previously reported polymorphism (rs35369693) [18] since there was no HapMap data available. The genotypes were obtained at an outsourced genotyping facility (The Centre for Applied Genomics Facility, Toronto, Canada) using the Sequenom MassArray® technology. Of the SNPs selected, rs11080121 and rs2066713 in *SLC6A4* as well as rs7124442 in *BDNF* could not be genotyped with this method. As a result, data from five SNPs were included in this study. At least 5% of the samples were genotyped twice and all duplicated genotypes were concordant. DNA samples of 8 patients had failed to be genotyped for all five polymorphisms investigated. Overall at least 94% of the patients were genotyped for each polymorphism. These polymorphisms, their minor allele frequencies (MAFs), and the successful genotyping rates are summarized in **Table 1**.

### Linkage Disequilibrium (LD) Map

The LD map of the *SLC6A4* gene (**Figure S1**) was constructed using the HapMap (phase I & II) genotype data obtained from the Caucasian samples (http://hapmap.ncbi.nlm.nih.gov/) using the Haploview program [17].

### Outcomes Investigated

The primary endpoint analyzed was overall survival (**OS**) defined as the time from date of diagnosis until the date of death from any cause. Our secondary endpoints were disease-free survival (DFS) and disease-specific survival (DSS). **DFS** is the time from diagnosis until the occurrence of first recurrence, metastasis or death from any cause. **DSS** is defined as the time from diagnosis until death due to colorectal cancer (or due to post-operative complications within the 30 days after surgery, n = 17). Patients who did not experience the event of interest were censored at the date of last follow up. Number of events for OS, DFS, and DSS are 162, 171, and 107, respectively.

### Demographic and Clinicopathological Variables

Disease stage, grade (poorly differentiated or undifferentiated vs well or moderately differentiated), location (rectum vs colon), age, sex (male vs female), histology (mucinous vs non-mucinous), and microsatellite stability status (microsatellite stable or microsatellite instability-low (MSS/MSI-L) vs microsatellite-instability high (MSI-H)) were investigated.

### Statistical Analysis

All genotypes were first manually analyzed to identify the major and the minor alleles and then were grouped together assuming the dominant inheritance model (aa+Aa vs AA, where a is the minor allele and A is the major allele). Deviation of the obtained genotype data from Hardy-Weinberg Equilibrium (HWE) was tested using an online tool described by Rodriguez and colleagues [19]. Age was analyzed as a continuous variable where as the remaining variables were categorized. We estimated the survival curves using the Kaplan-Meier method, which also provided the log-rank p-values. Differences between the survival times of different patient groups were examined using the Cox regression method, which calculated the p-values, hazard ratios (HRs), and 95% confidence intervals. Those variables that had p-values less than 0.05 in the univariate Cox regression analysis were entered into a multivariate analysis using the same method. In the genotype combination analysis for the *SLC6A4* SNPs, we combined patients with at least one minor allele (AG+AA genotypes in *SLC6A4*-rs4251417, CG+CC genotypes in *SLC6A4*-rs12150214, or AG+AA genotypes in *SLC6A4*-rs140700) and compared them with the patients homozygous for the major alleles of these polymorphisms.

In order to test whether the inclusion of the *SLC6A4* genotypes as a variable improved the predictive accuracy of the multivariate models, we calculated and compared the Akaike Information Criterion (AIC) [20] for three OS and DSS models. For both OS and DSS, Model 1 included age, stage, grade, and MSI status; Model 2 included the *SLC6A4*-rs12150214 genotype in addition to the variables in Model 1, and Model 3 included the combined *SLC6A4* genotypes in addition to the variables in Model 1. For each model, the AIC was calculated by the following formula: -2 log likelihood +2K, where -2 log likelihood is the likelihood of the Cox regression model and K is the number of parameters in the model for which an HR was calculated. The model with the smallest AIC represents the best informative model fitting the data.

Correlation between *SLC6A4*-combined genotypes and patient demographic and clinical parameters were tested using the Chi-square test. Statistical significance was set at p<0.05, all statistical tests were two-sided and were performed using the IBM-SPSS software package (version 19). Because of the hypothesis-generating nature of this study, correction for multiple testing was not done.

### Power Calculations

Power calculations for randomly selected hazard ratios of 2 and 1.5 were performed for each polymorphism using the Power and Sample Size program [21] assuming a follow up time of 11 years, accrual time of 2 years, and a type-I error probability (α) of 0.05. As a result, a minimum 80% study power was reached for each SNP to detect a hazard ratio of 2, but not 1.5. As expected, insufficient study power was more profound in less frequent polymorphisms (rs4251417 and rs140700 in *SLC6A4* and rs35369693 in *AVPR1B* genes) when compared to other two common polymorphisms (rs12150214 in *SLC6A4* and rs6265 in *BDNF*).

**Figure 1:**
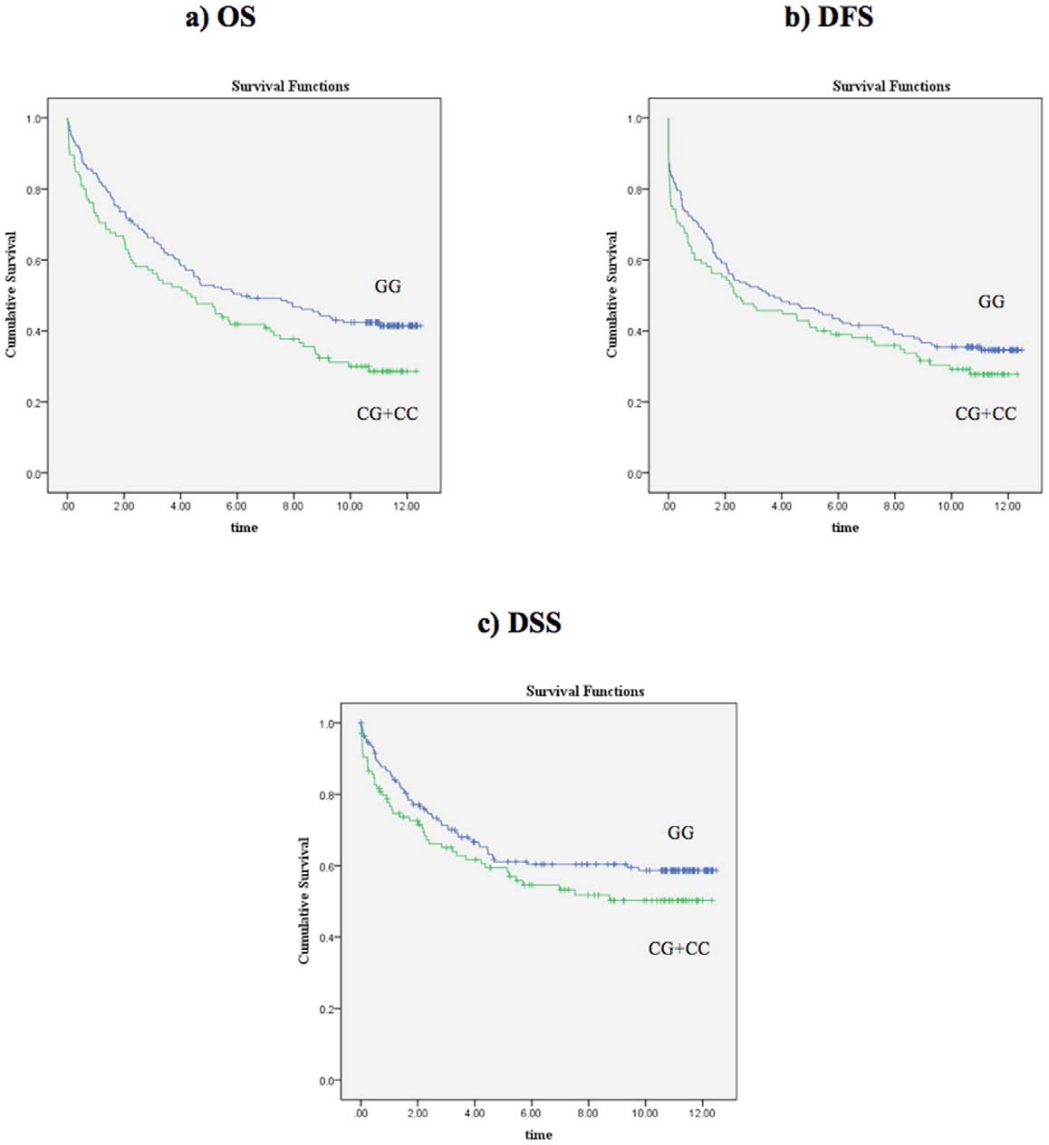
Kaplan-Meier survival curves for patients grouped based on the *SLC6A4*-rs12150214 genotype data. **a)** OS (p = 0.030, log-rank test), **b)** DFS (p = 0.225, log-rank test), and **c)** DSS (p = 0.159, log-rank test). Time is shown in years.

## Results

**Table 3:** Multivariate analysis results for OS.

			95% CI		
Variable	p-value	HR	Lower	Upper	n
*SLC6A4*-rs12150214 (CG+CC vs GG)	**.005**	**1.572**	**1.142**	**2.164**	259
Age	**<.001**	**1.049**	**1.034**	**1.064**	
Grade (poorly diff./undiff. vs well/moderately diff.)	**<.001**	**2.372**	**1.566**	**3.592**	
Stage	**<.001**				
Stage (II vs I)	**.715**	**1.103**	**.651**	**1.87**	
Stage (IIIvs I)	**.001**	**2.410**	**1.419**	**4.093**	
Stage (IV vs I)	**<.001**	**11.083**	**6.267**	**19.603**	
MSI status (MSI-H vs MSS/MSI-L)	**.002**	**.357**	**.186**	**.686**	

Only those variables with a p<0.05 in the univariate analysis are included in the multivariate analysis. Significant results are shown in bold. **CI:** confidence interval, **diff:** differentiated, **HR:** hazards ratio, **MSI-H:** microsatellite instability-high, **MSI-L:** microsatellite instability-low, **MSS:** microsatellite stable, **n**: number of samples included into the analysis, **vs:** versus. Number of events for OS is 162.

The baseline characteristics of this patient cohort are summarized in **Table 2**. As expected, the majority of the cases had well or moderately differentiated (i.e. low grade; 83.6%), non-mucinous (84.6%), colon (79.6%), and MSS/MSI-L (87.9%) tumors. The median age at diagnosis was 68.4 years. The five-year and ten-year overall survival rates for this cohort were 50% and 40%, respectively. Almost two-thirds of the patients had died (61.4%) or experienced disease progression (i.e. recurrence, metastasis, or death; 65.7%) by the last follow-up date. In addition, 40.4% of the cases had died of colorectal cancer (**Table 2**).

**Table 4:** Univariate analysis results for the combined genotypes of three SNPs within the *SLC6A4* gene.

			95% CI			
Variable	p-value	HR	Lower	Upper	n	Endpoint
*SLC6A4* variants (+ vs -)	**.005**	**1.544**	**1.137**	**2.097**	269	OS
*SLC6A4* variants (+ vs -)	.108	1.271	.949	1.704	269	DFS
*SLC6A4* variants (+ vs -)	**.009**	**1.656**	**1.133**	**2.419**	269	DSS

In the combined analysis, patients with at least one minor (variant) allele in any of the three SNPs in *SLC6A4* were categorized together (+) and were compared with the patients who did not have the variant alleles at these polymorphic loci (−). **OS:** overall survival, **DFS:** disease-free survival, **DSS:** disease-specific survival.

The minor allele frequencies (MAFs) of the polymorphisms are shown in **Table 1**. All polymorphisms were in Hardy-Weinberg Equilibrium. The univariate Cox regression analysis results for OS, DFS, and DSS are summarized in **Tables S1, S2, S3**. For the *SLC6A4*-rs12150214 polymorphism, patients carrying the variant C allele (CG or CC genotypes) were at increased risk of death from any cause (HR: 1.399, 95%CI: 1.031–1.897, p = 0.031) when compared to patients homozygous for the major allele (GG genotype) (**Figure 1**). However, a significant association of this SNP was not observed in either the DFS or the DSS analyses (**Figure 1**). Increasing age, disease stage, high tumor grade (poorly differentiated or undifferentiated), and MSS/MSI-L phenotype were also associated with worse OS, DFS, and DSS. Significant associations of the remaining four polymorphisms in *SLC6A4*, *BDNF*, and *AVPR1B* with OS, DFS, or DSS were not detected.

**Table 5:** Multivariate analysis results for the combined genotypes of three SNPs within the *SLC6A4* gene (OS).

			95% CI		
Variable	p-value	HR	Lower	Upper	n
*SLC6A4* variants (+ vs -)	**.002**	**1.631**	**1.190**	**2.236**	257
Age	**<.001**	**1.049**	**1.034**	**1.065**	
Grade (poorly diff./undiff. vs well/moderately diff.)	**<.001**	**2.364**	**1.556**	**3.589**	
Stage	**<.001**				
Stage (II vs I)	.698	1.111	.654	1.886	
Stage (IIIvs I)	**.001**	**2.476**	**1.456**	**4.211**	
Stage (IV vs I)	**<.001**	**11.244**	**6.335**	**19.957**	
MSI status (MSI-H vs MSS/MSI-L)	**.001**	**.331**	**.172**	**.640**	

Multivariate analysis results for overall survival. **(+):** patients with at least one minor (variant) allele in any of the three *SLC6A4* SNPs, **(−):** patients who did not have the variant allele in any of the three *SLC6A4* SNPs, **OS:** overall survival.

**Table 6:** Multivariate analysis results for the combined genotypes of three SNPs within the *SLC6A4* gene (DSS).

			95% CI		
Variable	p-value	HR	Lower	Upper	n
					
*SLC6A4* variants (+ vs -)	**.009**	**1.691**	**1.138**	**2.512**	257
Age	**<.001**	**1.038**	**1.020**	**1.056**	
Grade (poorly diff./undiff. vs well/moderately diff.)	**<.001**	**2.917**	**1.798**	**4.733**	
Stage	**<.001**				
Stage (II vs I)	.154	2.040	.766	5.436	
Stage (IIIvs I)	**<.001**	**6.630**	**2.573**	**17.083**	
Stage (IV vs I)	**<.001**	**35.993**	**13.77**	**94.097**	
MSI status (MSI-H vs MSS/MSI-L)	**.002**	**.207**	**.075**	**.574**	

Multivariate analysis results for disease-specific survival. **(+):** patients with at least one minor (variant) allele in any of the three *SLC6A4* SNPs, **(−):** patients who did not have the variant allele in any of the three *SLC6A4* SNPs, **DSS:** disease-specific survival.

**Table 7:** Akaike information criterion (AIC) calculations for the OS and DSS multivariate models with or without the *SLC6A4* genotypes.

Endpoint	−2 log likelihood	Number of parameters	AIC	ΔAIC
**OS**				
Model 1	1545.859	6	1557.859	53.792
Model 2	1514.252	7	1528.252	24.185
Model 3	1490.067	7	1504.067	0
**DSS**				
Model 1	977.655	6	989.655	30.834
Model 2	962.69	7	976.69	17.869
Model 3	944.821	7	958.821	0

Model 1 contains the clinical variables that were significant in the multivariate analyses; namely age, stage, grade, and MSI-H status (for both OS and DSS); Model 2 contains the *SLC6A4*-rs12150214 genotype as a variable in addition to the clinical variables in Model 1, and Model 3 contains the combined *SLC6A4* genotypes (for rs4251417, rs12150214, rs140700) in addition to the variables in Model 1. ΔAIC is the difference in AIC values of a model and the best model, which is Model 3 for both OS and DSS.

**Figure 2:**
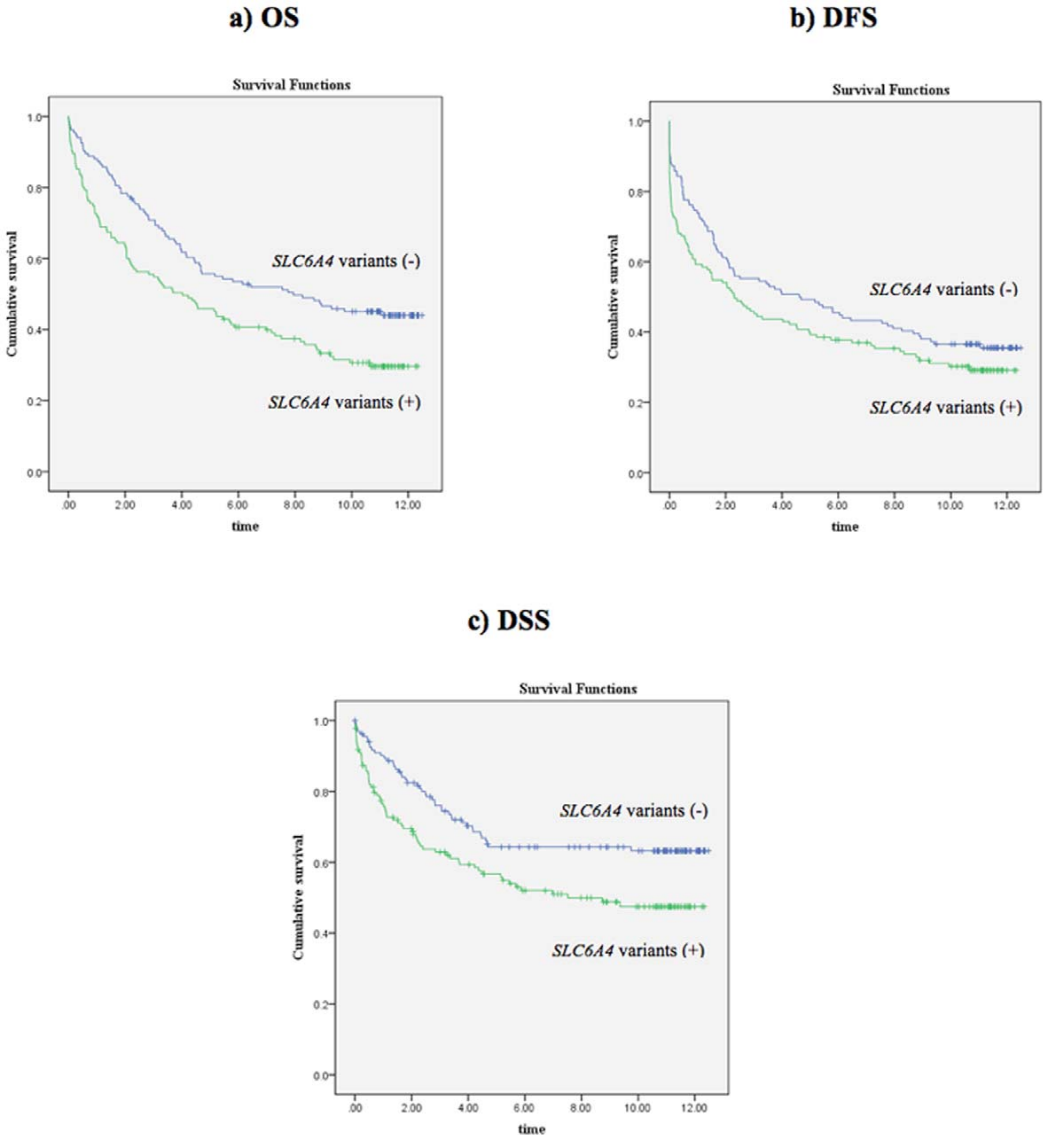
Kaplan-Meier survival curves for the combined genotypes of three *SLC6A4* polymorphisms. **a)** OS (p = 0.005, log-rank test), **b)** DFS (p = 0.104, log-rank test), and **c)** DSS (p = 0.008, log-rank test). Time is shown in years.

The results of the multivariate analysis for OS are shown in **Table 3**. Similar to the univariate analysis, for the *SLC6A4*-rs12150214 polymorphism, when compared to the patients with major homozygote genotype (GG), patients carrying the variant C allele (CG or CC genotypes) were at increased risk of death from any cause (HR: 1.572, 95%CI: 1.142–2.164, p = 0.005), after adjustment for sex, age, grade, stage and MSI status. Increasing age, grade, stage, and MSI status were also independent predictors of OS in this cohort (**Table 3**). Additional multivariate analyses also showed the independent association of age, grade, stage, and MSI status with both DFS and DSS (*data not shown*).

We also performed a genotype combination analysis for the three SNPs from the *SLC6A4* gene. Patients with genotypes with at least one minor allele in any of the three SNPs were compared with patients who were homozygous for major alleles. Univariate analysis showed that the presence of at least one minor allele was associated with worse OS and DSS, but not with DFS in our cohort (**Table 4** **;** **Figure 2**). More interestingly, when adjusted for other variables, the presence of at least one minor allele was independently associated with both worse OS (HR: 1.631, 95%CI: 1.190–2.236, p = 0.002) and worse DSS (HR: 1.691, 95%CI: 1.138–2.512, p = 0.009) (**Tables 5** **and** **6**). These associations were stronger than those detected when *SLC6A4*-rs12150214 was analyzed alone (**Table 3** **;** **Figures 1** **and** **2**). The AIC calculations indicated that the addition of the *SLC6A4*-rs12150214 genotype into the multivariate models (both for OS and DSS) also containing age, stage, grade, and MSI status variables improved the model predictions (**Table 7**). However, the best improvement was detected when the model contained the combined *SLC6A4* genotypes in addition to the clinical variables (**Table 7**). In addition, when compared to the DSS model, the model improvement was more profound in the OS model.

There was no correlation between the combined genotype status and the baseline demographic and clinical characteristics listed in **Table 2** (*data not shown*).

**Figure 3:**
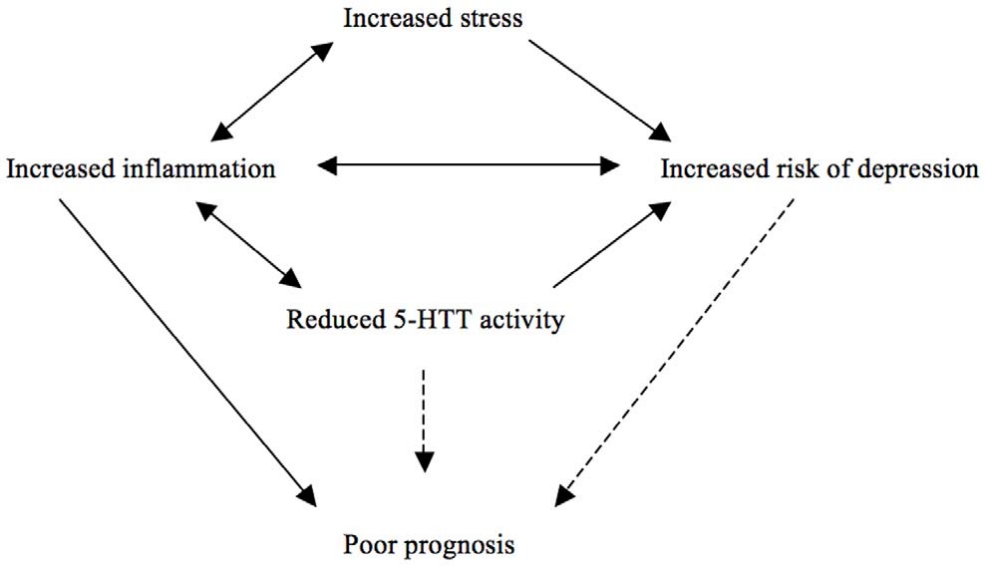
The 5-HTT activity may be altered as a response to changing cellular environment (such as inflammation) or by the genetic variations in the *SLC6A4* gene. The direct links between the altered 5-HTT activity as well as the depression and prognosis in colorectal cancer patients (arrows with broken lines) remain to be established by further studies.

## Discussion

While well-established prognostic markers such as disease stage are used in prognostication, an inter-patient prognostic variability remains [3]. Better prognostic performance may come from the identification of yet-to-be recognized prognostic indicators. For example, several epidemiological studies have suggested an effect of life-style factors (such as level of physical activity) on prognosis [22], [23]. Although not well studied in colorectal cancer, emotional distress and psychological interventions after diagnosis have been implicated in modification of outcome [24]–[27]. Consequently, in the present study, we have aimed to test the associations of five genetic variations in stress response genes (*SLC6A4, BDNF,* and *AVPR1B*) with clinical outcomes in a population-based cohort of 280 colorectal cancer patients from Newfoundland and Labrador (NL). NL is one of the 13 provinces/territories in Canada and has the highest incidence of colorectal cancer as well as one of the highest cancer mortality rates in Canada [1], [28]. This population is also characterized by a high incidence rate of familial colorectal cancer [29], [30]. A strength of our study is the long period of time that patients have been followed (up to 12.5 years after cancer diagnosis). As a consequence we have documented many events, which increased our study power. A weakness of our study is that the results obtained remain to be replicated in other cohorts. In addition, serum biomarkers with prognostic potentials, such as interleukin-6 (IL-6), carcinoembryonic antigen (CEA), and carbohydrate antigen 19–9 (CA 19–9) were not evaluated in our study.

Our results showed that the polymorphisms in neither the *BDNF* nor the *AVPR1B* genes were correlated with outcome in our patient cohort, though we cannot rule out the false-negative findings due to lack of adequate study power, especially in the case of the relatively rare *AVPR1B-*rs35369693 polymorphism. However, we found that the rs12150214 SNP, which is a relatively common G>C substitution in the non-coding region of the *SLC6A4* gene, was associated with poor OS in a univariate as well as in a multivariate analysis. Specifically, when adjusted for sex, stage, grade, age and MSI status, the patients carrying the variant C allele (both CC homozygotes and CG heterozygotes) were at 57% increased risk of death (95%CI: 1.142–2.164; **Table 3**) when compared to patients with the GG genotype. When the genotypes containing the minor alleles were combined for the three *SLC6A4* SNPs, we detected a significant correlation with not only OS, but also with DSS (**Tables 5** **and** **6**). These associations were independent of other clinically important variables including age, stage, grade, and MSI status. Although our approach did not include all variations in the *SLC6A4* gene, these results suggest the association of multiple *SLC6A4* variations with prognosis in colorectal cancer patients. In addition, our results show that inclusion of the *SLC6A4* genotypes, particularly the combined genotypes, as a variable improves the predictive accuracy of both the OS and DSS multivariate models (**Table 7**). **Figures 1** **and** **2** also show that the combined *SLC6A4* genotypes, when compared to rs12150214 genotypes alone, results in a better separation of patient survival curves.

The linkage disequilibrium (LD) map of the *SLC6A4* gene and the relative positions of the three SNPs investigated in the present study (rs4251417, rs12150214, and rs140700) are shown in **Figure S1**. These three SNPs are all non-coding polymorphisms and are located in non-linked regions of the *SLC6A4* gene (i.e. they are not correlated with each other; r^2^<0.8). Specifically, rs4251417 and rs12150214 are located in the 5′-end of the gene (in intron 1) and rs140700 is located in intron 6. At the time being, it is not clear how these polymorphisms may affect the *SLC6A4* gene expression or function; however, it is also likely that other functional polymorphisms that are highly correlated with them in fact might be the variants biologically related to altered gene function and thus prognosis (**Figure S1**).

To our knowledge, there is no report linking rs4251417 to stress response or other human disease. However, previously, the rs12150214 polymorphism (located in the LD block-2 in **Figure S1**) was reported to be borderline associated with the Beck Depression Inventory scores in a study investigating 360 male twins [31]. These authors also performed a haplotype analysis with the polymorphisms linked to rs12150214 and found that this region of the *SLC6A4* gene was associated with not only depression but also elevated IL-6 levels in the study subjects. Interestingly, IL-6 is a pro-inflammatory cytokine (*see below*). In another study involving 567 subjects, Lazary *et al*. [32] suggested that the rs140700 polymorphism (or other variations linked to it) acts as a modifier of depressive symptoms (this polymorphism is located in the LD block-1 in **Figure S1**). These previously reported findings suggest that multiple variations within the *SLC6A4* gene are likely to be involved in altering gene function with adverse consequences for stress response and depression. This can also explain why we have detected a stronger association with both OS and DSS in our patient cohort when the genotype data for the three *SLC6A4* SNPs were combined and analyzed in the multivariate analyses. However, in the absence of the psychological health data in our cohort, we cannot determine whether the observed association of the *SLC6A4* gene with shorter survival times is due to its role in psychological distress or due to other biological roles of the *SLC6A4*.

In fact, in addition to its role in psychological stress response, the 5-HTT protein coded by the *SLC6A4* gene as well as its ligand (serotonin) function in the immune response and inflammation, including in gut. For example, serotonin signaling regulates stool transition in the gut and the serotonin levels in this organ may be altered (either by altered rates of serotonin secretion or function of the 5-HTT protein) as a result of local infection and inflammation [33]. Interestingly, a recent study using a mouse model of colitis showed that the loss of 5-HTT activity enhances the severity of inflammation in the colon [34]. These findings suggest the presence of a two-way interaction between 5-HTT and inflammation. A similar pattern is also observed in inflammation and depression, where the one seems to induce or exacerbate the other [35], [36]. Increased or persistent inflammation is also linked to tumor progression, including colorectal cancers [37], [38]. Interestingly IL-6 was previously reported to be associated with variations in the *SLC6A4* gene [31] (*see the previous paragraph*), which also seems to be a mediator of tumor progression in colorectal cancer [37], [39], [40]. Therefore, we suggest that emotional or physiological stress (i.e. inflammation), and their associated neurological and immune responses can be modified by the activity of the 5-HTT protein (either by environmental factors or by the genetic variations in the *SLC6A4* gene), which can explain the poor survival observed in our cohort of colorectal cancer patients (**Figure 3**). Further studies on the potential roles of the *SLC6A4* gene variations and the depression in survival of colorectal cancer patients are therefore warranted.

## Supporting Information

Figure S1 The three *SLC6A4* SNPs investigated in this study (rs4251417, rs12150214, and rs140700) are circled. The black triangles designate the LD blocks. The red squares are where the correlation between markers is the strongest. Please note that the gene is shown in a 3′ to 5′ orientation in this figure. According to the Haploview (pairwise tagger) [17] results, rs12150214 is highly correlated with the following polymorphisms along the *SLC6A4* gene with a correlation coefficient (r^2^) of >0.85: rs8076005, rs11080122, rs6354, rs25528, rs2020936, and rs8071667 (annotated with stars on the **Figure S1**).
(TIFF)

Table S1 Significant results are shown in bold. **CI:** confidence interval, **diff:** differentiated, **HR:** hazards ratio, **MSI-H:** microsatellite instability-high, **MSI-L:** microsatellite instability-low, **MSS:** microsatellite stable, **n**: number of samples included into the analysis, **vs:** versus.
(DOC)

Table S2 Significant results are shown in bold. **CI:** confidence interval, **diff:** differentiated, **HR:** hazards ratio, **MSI-H:** microsatellite instability-high, **MSI-L:** microsatellite instability-low, **MSS:** microsatellite stable, **n**: number of samples included into the analysis, **vs:** versus.
(DOC)

Table S3 Significant results are shown in bold. **CI:** confidence interval, **diff:** differentiated, **HR:** hazards ratio, **MSI-H:** microsatellite instability-high, **MSI-L:** microsatellite instability-low, **MSS:** microsatellite stable, **n**: number of samples included into the analysis, **vs:** versus.
(DOC)
