# Supplementary figures and images for: Serotonin Transporter Gene (SLC6A4) Variations Are Associated with Poor Survival in Colorectal Cancer Patients
Source: PLoS One. 2012 Jul 24;7(7):e38953. doi: 10.1371/journal.pone.0038953 (PMC3404081; doi:10.1371/journal.pone.0038953)

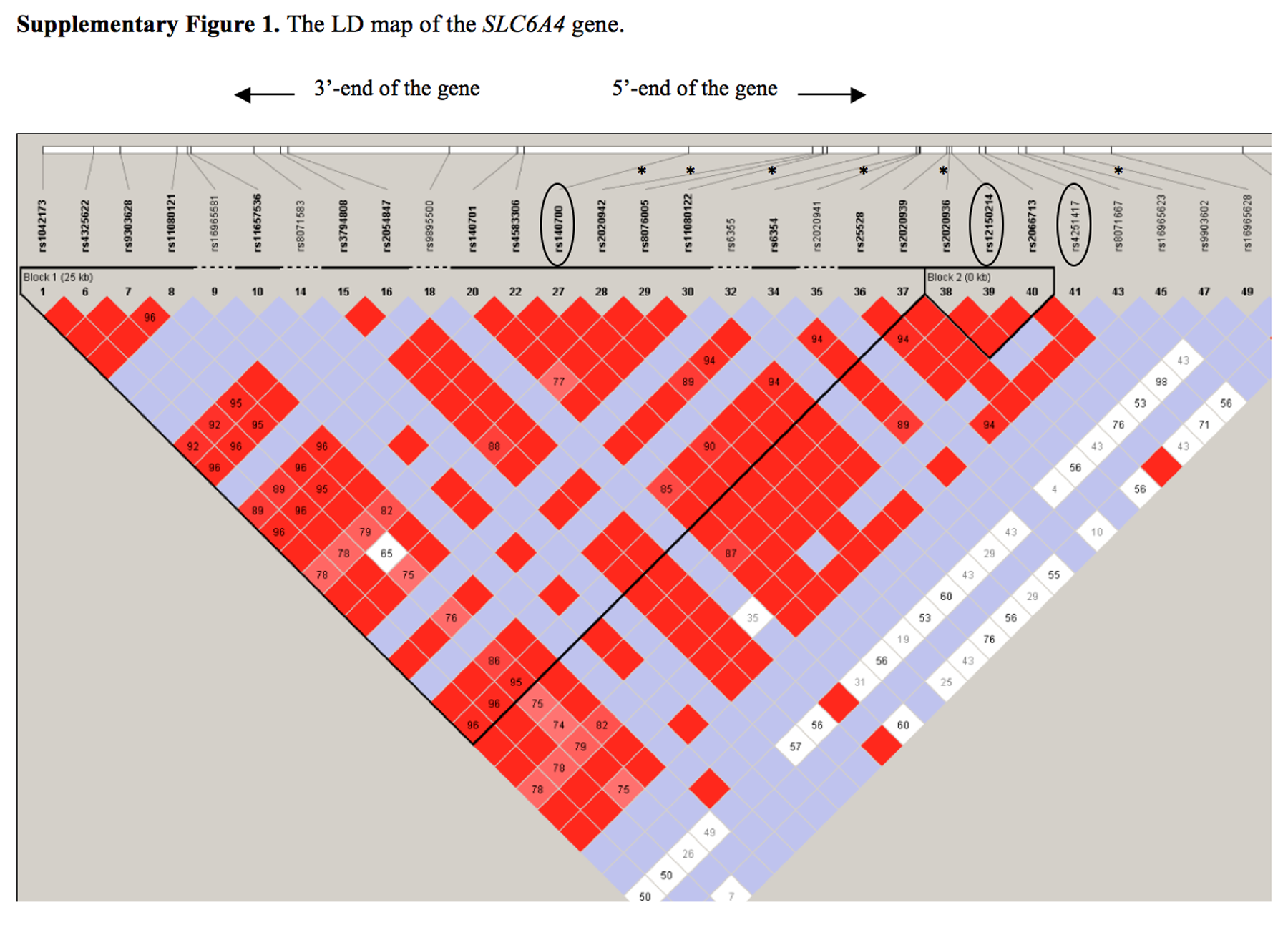

Supplement: Figure S1 — The three SLC6A4 SNPs investigated in this study (rs4251417, rs12150214, and rs140700) are circled. The black triangles designate the LD blocks. The red squares are where the correlation between markers is the strongest. Please note that the gene is shown in a 3′ to 5′ orientation in this figure. According to the Haploview (pairwise tagger) [17] results, rs12150214 is highly correlated with the following polymorphisms along the SLC6A4 gene with a correlation coefficient (r2) of >0.85: rs8076005, rs11080122, rs6354, rs25528, rs2020936, and rs8071667 (annotated with stars on the Figure S1). (TIFF) [file pone.0038953.s001.tiff]
