# Supplementary material for: Serotonin Transporter Gene (SLC6A4) Variations Are Associated with Poor Survival in Colorectal Cancer Patients
Source: PLoS One. 2012 Jul 24;7(7):e38953. doi: 10.1371/journal.pone.0038953 (PMC3404081; doi:10.1371/journal.pone.0038953)
Supplement: Table S1 — Significant results are shown in bold. CI: confidence interval, diff: differentiated, HR: hazards ratio, MSI-H: microsatellite instability-high, MSI-L: microsatellite instability-low, MSS: microsatellite stable, n: number of samples included into the analysis, vs: versus. (DOC) [file pone.0038953.s002.doc]

**Table S1.** Univariate analysis results for OS.

|  |  |  | **95% CI** | |  |
| --- | --- | --- | --- | --- | --- |
| **Variables** | **p-value** | **HR** | **Lower** | **Upper** | **n** |
|  |  |  |  |  |  |
| *SLC6A4*-rs4251417 (AG+AA vs GG) | .392 | 1.194 | 0.796 | 1.79 | 272 |
| *SLC6A4*-rs12150214 (CG+CC vs GG) | **.031** | **1.399** | **1.031** | **1.897** | 271 |
| *SLC6A4*-rs140700 (AG+AA vs GG) | .502 | 1.143 | 0.774 | 1.689 | 268 |
| *BDNF*-rs6265 (AG+AA vs GG) | .969 | 1.006 | 0.733 | 1.381 | 271 |
| *AVPR1B*-rs35369693 (CG+CC vs GG) | .317 | 1.247 | 0.809 | 1.922 | 264 |
| Sex (male vs female) | .094 | 1.296 | 0.957 | 1.757 | 280 |
| Age | **<.001** | **1.034** | **1.021** | **1.047** | 280 |
| Grade (poorly diff./undiff. vs well/moderately diff.) | **.001** | **1.949** | **1.325** | **2.866** | 276 |
| Histology (mucinous vs non-mucinous) | .603 | 1.113 | 0.743 | 1.669 | 280 |
| Location (rectum vs colon) | .343 | 1.191 | 0.83 | 1.708 | 280 |
| Stage | **<.001** |  |  |  | 271 |
| Stage (II vs I) | .150 | 1.458 | 0.873 | 2.435 |  |
| Stage (III vs I) | **<.001** | **2.464** | **1.483** | **4.094** |  |
| Stage (IV vs I) | **<.001** | **9.647** | **5.629** | **16.534** |  |
| MSI status (MSI-H vs MSS/MSI-L) | **.001** | **0.359** | **0.195** | **0.662** | 280 |
