# Supplementary material for: Serotonin Transporter Gene (SLC6A4) Variations Are Associated with Poor Survival in Colorectal Cancer Patients
Source: PLoS One. 2012 Jul 24;7(7):e38953. doi: 10.1371/journal.pone.0038953 (PMC3404081; doi:10.1371/journal.pone.0038953)
Supplement: Table S2 — Significant results are shown in bold. CI: confidence interval, diff: differentiated, HR: hazards ratio, MSI-H: microsatellite instability-high, MSI-L: microsatellite instability-low, MSS: microsatellite stable, n: number of samples included into the analysis, vs: versus. (DOC) [file pone.0038953.s003.doc]

**Table S2.** Univariate analysis results for DFS.

|  |  |  | **95% CI** | |  |
| --- | --- | --- | --- | --- | --- |
| **Variables** | **p-value** | **HR** | **Lower** | **Upper** | **n** |
|  |  |  |  |  |  |
| *SLC6A4*-rs4251417 (AG+AA vs GG) | .633 | 1.103 | 0.737 | 1.65 | 272 |
| *SLC6A4*-rs12150214 (CG+CC vs GG) | .230 | 1.199 | 0.892 | 1.612 | 271 |
| *SLC6A4*-rs140700 (AG+AA vs GG) | .576 | 1.115 | 0.761 | 1.634 | 268 |
| *BDNF*-rs6265 (AG+AA vs GG) | .916 | 0.984 | 0.726 | 1.334 | 271 |
| *AVPR1B*-rs35369693 (CG+CC vs GG) | .207 | 1.307 | 0.862 | 1.981 | 264 |
| Sex (male vs female) | .264 | 1.181 | 0.882 | 1.579 | 280 |
| Age | **<.001** | **1.025** | **1.013** | **1.037** | 280 |
| Grade (poorly diff./undiff. vs well/moderately diff.) | **.009** | **1.659** | **1.136** | **2.422** | 276 |
| Histology (mucinous vs non-mucinous) | .678 | 1.086 | 0.735 | 1.607 | 280 |
| Location (rectum vs colon) | .288 | 1.205 | 0.854 | 1.701 | 280 |
| Stage | **<.001** |  |  |  | 271 |
| Stage (II vs I) | .057 | 1.618 | 0.985 | 2.657 |  |
| Stage (III vs I) | **<.001** | **2.756** | **1.678** | **4.525** |  |
| Stage (IV vs I) | **<.001** | **102.4** | **47.03** | **222.87** |  |
| MSI status (MSI-H vs MSS/MSI-L) | **<.001** | **0.348** | **0.194** | **0.625** | 280 |
