# Supplementary material for: Serotonin Transporter Gene (SLC6A4) Variations Are Associated with Poor Survival in Colorectal Cancer Patients
Source: PLoS One. 2012 Jul 24;7(7):e38953. doi: 10.1371/journal.pone.0038953 (PMC3404081; doi:10.1371/journal.pone.0038953)
Supplement: Table S3 — Significant results are shown in bold. CI: confidence interval, diff: differentiated, HR: hazards ratio, MSI-H: microsatellite instability-high, MSI-L: microsatellite instability-low, MSS: microsatellite stable, n: number of samples included into the analysis, vs: versus. (DOC) [file pone.0038953.s004.doc]

**Table S3.** Univariate analysis results for DSS.

|  |  |  | **95% CI** | |  |
| --- | --- | --- | --- | --- | --- |
| **Variables** | **p-value** | **HR** | **Lower** | **Upper** | **n** |
|  |  |  |  |  |  |
| *SLC6A4*-rs4251417 (AG+AA vs GG) | .118 | 1.451 | 0.91 | 2.313 | 272 |
| *SLC6A4*-rs12150214 (CG+CC vs GG) | .160 | 1.31 | 0.899 | 1.909 | 271 |
| *SLC6A4*-rs140700 (AG+AA vs GG) | .385 | 1.23 | 0.771 | 1.963 | 268 |
| *BDNF*-rs6265 (AG+AA vs GG) | .809 | 0.953 | 0.644 | 1.409 | 271 |
| *AVPR1B*-rs35369693 (CG+CC vs GG) | .277 | 1.332 | 0.794 | 2.234 | 264 |
| Sex (male vs female) | .246 | 1.248 | 0.858 | 1.814 | 280 |
| Age | **.029** | **1.017** | **1.002** | **1.032** | 280 |
| Grade (poorly diff./undiff. vs well/moderately diff.) | **<.001** | **2.333** | **1.493** | **3.647** | 276 |
| Histology (mucinous vs non-mucinous) | .774 | 0.926 | 0.545 | 1.571 | 280 |
| Location (rectum vs colon) | .520 | 1.157 | 0.742 | 1.805 | 280 |
| Stage | **<.001** |  |  |  | 271 |
| Stage (II vs I) | **.067** | **2.324** | **0.942** | **5.731** |  |
| Stage (III vs I) | **<.001** | **6.067** | **2.562** | **14.364** |  |
| Stage (IV vs I) | **<.001** | **28.08** | **11.78** | **66.917** |  |
| MSI status (MSI-H vs MSS/MSI-L) | **.002** | **0.203** | **0.075** | **0.551** | 280 |
